# Improving efficiency in financial process of the National Health Security Scheme

**DOI:** 10.1186/1471-2458-14-S1-P24

**Published:** 2014-01-29

**Authors:** Niramol Henprasert, Supasit Pannarunothai, Nilawan Upakdee, Pudtan Phanthunane

**Affiliations:** 1 National Health Security Office, Nonthaburi 11000, Thailand; 2 Faculty of Medicine, Naresuan University, Phitsanulok 65000, Thailand; 3 Faculty of Pharmaceutical Sciences, Naresuan University, Phitsanulok 65000, Thailand; 4 Faculty of Business, Economics and Communications, Naresuan University, Phitsanulok 65000, Thailand

## Background

The National Health Security Scheme was established 10 years ago with the document finance model in communicating the transfer of budget from payer to providers. This model was found to be inefficient in transferring (17 billion Baht delay) or not knowing the amount transferred (33 billion Baht bad debt). A new ageing account model was developed 3 years ago to improve efficiency in financial process. It is interesting to know whether the change achieved the efficiency. This research also wanted to compare efficiency in financial process of all three government health insurance funds managed by the National Security Health Office (NHSO managing the universal coverage scheme), the Social Security Office (SSO managing insurance for workers in private sector) and the Comptroller General Department (CGD managing insurance for civil servants and dependents).

## Materials and methods

This research employed two rounds of questionnaire surveys to heads of finance department in hospitals in 2010 and 2012. An in-depth interview was employed in ten of responses from the Ministry of Public Health hospitals commenting that the new model achieved the most efficient financial process and another ten complaining the least efficient process. Eight hospitals outside the MOPH were added to get unbiased views comparing financial processes of the three government health insurance funds. The interviews were undertaken from November 2012 to February 2013.

## Results

Comparing results from the two surveys: providers felt a marginal increase in financial process of the ageing account model in terms of efficient transfer, outstanding realisation, speed and the ease of use of transferred fund. The new model was inferior to the old model in terms of the frequency of sending financial report. Qualitative data confirmed that efficiency was interpreted as the quickness and correctness of fund transfer. The better efficient responders explained that the NHSO transferred fund became quicker and simpler to use. However, the least efficient responders explained that the CGD transferred fund quicker than the NHSO and used web-based financial report for quickness and transparency. The accounting items of the NHSO were the most difficult to understand.

## Conclusions

With perceived marginal increase in efficiency of the new account model, the NHSO should improve timeliness and comprehensiveness of the account model as a benchmark with other two government health insurance funds.

